# Association Between Maternal Syphilis Infection and Stillbirth Among Women Delivering at Jinja and Hoima Regional Referral Hospitals: An Unmatched Case–Control Study

**DOI:** 10.1155/ogi/6882011

**Published:** 2026-09-12

**Authors:** Daniel Mugabi, Theoneste Hakizimana, Musa Kasujja, John Yaweh, Sande George Bina, Adam Eibad, Geoffrey Okot, Neel Rajendra, Emmanuel Okurut, Dominic Byarugaba, Ronard Musinguzi

**Affiliations:** ^1^ Kampala International University, Kampala, Uganda, kiu.ac.ug; ^2^ Gulu University, Gulu, Uganda, gu.ac.ug

## Abstract

**Background and Aims:**

Maternal syphilis infection remains a preventable contributor to stillbirth in low‐ and middle‐income countries. Evidence on its distribution among stillbirth deliveries, its independent association with fetal death, and the role of infection severity remains limited in Ugandan referral hospitals. This study aimed to compare maternal syphilis infection between stillbirth and live‐birth deliveries, estimate its adjusted association with stillbirth, and assess whether maternal rapid plasma reagin (RPR) titers showed a dose–response relationship with stillbirth.

**Methods:**

A hospital‐based unmatched case–control study was conducted among 406 women delivering at Jinja and Hoima Regional Referral Hospitals from January to April 2026. Maternal syphilis infection was determined using serologic testing. Group differences were assessed using chi‐square tests, and multivariable logistic regression estimated adjusted odds ratios (AORs) and 95% confidence intervals (CIs).

**Results:**

Maternal syphilis infection was more common among stillbirth deliveries than live births (11.9% vs. 3.7%; *χ* ^2^ (1) = 10.015, *p* = 0.002). After adjustment, maternal syphilis infection was associated with higher odds of stillbirth (AOR 3.91, 95% CI: 1.65–9.31; *p* = 0.002). Increasing RPR titer category was associated with increasing odds of stillbirth (ordinal trend OR 1.98, 95% CI: 1.26–3.12; *p* = 0.003).

**Conclusion:**

Maternal syphilis infection was independently associated with stillbirth, and higher RPR titer categories showed a graded association with stillbirth. These findings support strengthened antenatal syphilis screening, timely treatment, and risk‐based follow‐up in referral‐hospital settings.

## 1. Introduction

Stillbirth remains a major global public health challenge, with the burden disproportionately concentrated in low‐ and middle‐income countries, particularly in sub‐Saharan Africa. Globally, untreated maternal syphilis has been estimated to account for more than 200,000 stillbirths and early fetal deaths annually, with the majority occurring in settings where antenatal screening and treatment coverage remain incomplete [1]. Systematic reviews and global modeling studies consistently demonstrate that approximately one in five pregnancies complicated by untreated maternal syphilis results in fetal loss or stillbirth, underscoring the substantial preventable burden attributable to this infection.

Maternal syphilis contributes to adverse pregnancy outcomes through well‐established biological mechanisms, including placental inflammation, impaired uteroplacental perfusion, transplacental transmission of Treponema pallidum, and subsequent fetal compromise. These mechanisms substantially increase the risk of stillbirth, preterm birth, and neonatal death. Meta‐analytic evidence indicates that untreated maternal syphilis infection is associated with markedly higher risks of adverse pregnancy outcomes compared with syphilis‐seronegative pregnancies, while timely antenatal detection and benzathine penicillin treatment can substantially reduce stillbirth risk, highlighting the effectiveness of screening‐based prevention strategies in routine maternal care [2, 3]. Vertical transmission risk varies by stage of maternal infection and is highest in early syphilis; reported transmission rates are approximately 60%–100% in primary or secondary syphilis, about 40% in early latent infection, and lower in late latent infection [4].

Evidence from African hospital‐based studies and regional syntheses consistently demonstrates a higher burden of maternal syphilis infection among pregnancies ending in stillbirth or other adverse outcomes than among unaffected pregnancies. Recent evidence from Malawi reported that sexually transmitted infections in pregnancy were more frequent among adverse birth outcome cases than healthy controls in a referral‐hospital setting [5], while data from Tanzania showed persistent syphilis seropositivity among women presenting with macerated stillbirth, particularly in the presence of HIV co‐infection and rural residence [6]. Regional reviews from sub‐Saharan Africa similarly report positive associations between Treponema pallidum infection and stillbirth across multiple observational studies, supporting the continued relevance of maternal syphilis as a determinant of fetal mortality in high‐burden African settings [7].

More recent evidence has emphasized the importance of infection severity, particularly maternal rapid plasma reagin (RPR) antibody titers, as a marker of disease activity and predictor of adverse pregnancy outcomes. Large retrospective analyses from national prevention‐of‐mother‐to‐child‐transmission programs have demonstrated that maternal titers greater than 1:4 significantly increase the odds of stillbirth, even after adjustment for maternal demographic and clinical characteristics. Similarly, population‐based studies have demonstrated progressive increases in adverse pregnancy outcomes across increasing titer categories, supporting the presence of a biologically plausible dose–response relationship between infection severity and fetal compromise [8, 9].

Regional systematic reviews further confirm that untreated maternal syphilis remains a leading preventable cause of fetal loss and stillbirth in sub‐Saharan Africa, with observational studies showing consistently elevated effect estimates and pooled analyses demonstrating large absolute differences in stillbirth risk between infected and uninfected pregnancies. Contemporary evidence also indicates that timely antibiotic treatment during pregnancy substantially reduces the risk of stillbirth, emphasizing the importance of strengthening screening programs in referral‐hospital populations where late presentation remains common [7, 10, 11].

In Uganda, referral hospitals continue to report stillbirth rates exceeding national targets, with maternal infections identified as key contributors to this burden. Recent facility‐based evidence from western Uganda has demonstrated substantial missed opportunities for antenatal syphilis testing and a high prevalence of maternal infection among women not screened during pregnancy, underscoring persistent gaps in prevention coverage [12]. Jinja and Hoima Regional Referral Hospitals serve large catchment populations and manage a high proportion of complicated pregnancies referred from peripheral facilities. Despite routine antenatal screening for syphilis in these facilities, evidence describing the distribution of maternal syphilis infection among stillbirth deliveries, its independent association with fetal death after adjustment for major maternal and obstetric confounders, and the role of infection severity measured using RPR titers remains limited.

Furthermore, few hospital‐based studies in Uganda have evaluated whether increasing maternal RPR titers demonstrate a biological gradient consistent with increasing stillbirth risk after adjustment for major maternal and obstetric confounders. Demonstrating such a dose–response relationship is important for strengthening causal inference, improving clinical risk stratification during antenatal care, and informing targeted prevention strategies in high‐burden referral‐hospital settings.

Maternal syphilis represents one of the few infectious causes of stillbirth for which the causal pathway, diagnostic identification, and effective treatment strategy are well established, making it a priority target for prevention within antenatal care programs and global congenital syphilis elimination initiatives [11, 13]. Because maternal infections such as HIV and malaria frequently co‐occur with syphilis in East African referral‐hospital populations and independently contribute to stillbirth risk, adjustment for these conditions is essential when estimating the independent effect of maternal syphilis infection on fetal mortality.

However, evidence from multivariable‐adjusted referral‐hospital case–control studies evaluating whether increasing maternal RPR titers demonstrate a biological gradient in stillbirth risk remains limited in Uganda. Identifying whether maternal RPR titers demonstrate a graded association with stillbirth risk may support the use of serological severity markers for antenatal risk stratification in high‐burden referral‐hospital settings.

Therefore, this study aimed to estimate the difference in maternal syphilis infection prevalence between stillbirth and live‐birth deliveries, quantify the adjusted association between maternal syphilis infection and stillbirth, and evaluate whether increasing maternal RPR titers demonstrate a biological gradient consistent with increasing stillbirth risk among women delivering at Jinja and Hoima Regional Referral Hospitals in Uganda.

## 2. Materials and Methods

### 2.1. Study Design and Setting

#### 2.1.1. Study Design

This study employed a hospital‐based unmatched case–control design to investigate the association between maternal syphilis infection and stillbirth among deliveries at two regional referral hospitals in Uganda. The study design and analytical strategy were guided by an a priori causal framework informed by epidemiological evidence linking maternal infection, placental pathology, and adverse perinatal outcomes.

#### 2.1.2. Study Setting

The study was conducted at Jinja Regional Referral Hospital (Eastern Uganda) and Hoima Regional Referral Hospital (Western Uganda), both government‐funded tertiary‐level facilities providing comprehensive obstetric and neonatal services, including routine antenatal screening for sexually transmitted infections. These hospitals serve large referral catchment populations of approximately 2.8 million and 1.5 million people, respectively. Data collection was conducted between January and April 2026.

At both hospitals, syphilis screening is provided as part of routine antenatal care, with testing usually performed at the first antenatal contact or when women present for delivery without documented antenatal results. Repeat RPR testing during pregnancy was not consistently documented in the records available for this study. Women with reactive syphilis results were expected to receive treatment according to routine facility practice, with counselling on partner notification and treatment. For stillbirth deliveries, families received routine post‐delivery counselling and clinical review, including consideration of maternal infections and other obstetric contributors where records were available.

#### 2.1.3. Study Population

The study population comprised postpartum women aged 15–49 years who delivered at ≥ 26 weeks of gestation at Jinja and Hoima Regional Referral Hospitals during the study period. Cases were defined as women who delivered stillborn infants, whereas controls were women who delivered live‐born infants.

#### 2.1.4. Eligibility Criteria

##### 2.1.4.1. Inclusion Criteria

Participants were eligible if they

• delivered at either Jinja or Hoima Regional Referral Hospital during the study period;
• had a gestational age of ≥ 26 weeks at delivery; and
• provided written informed consent for participation.

##### 2.1.4.2. Exclusion Criteria

Participants were excluded if they

• had multiple gestation pregnancies;
• had incomplete or missing laboratory records for syphilis diagnosis or RPR titers; or
• had incomplete antenatal or intrapartum records for key covariates, including HIV status, malaria status, hypertensive disorders of pregnancy, or antenatal care attendance.

### 2.2. Data Collection Procedure

#### 2.2.1. Sampling and Recruitment Procedure

All eligible stillbirth deliveries occurring during the study period were consecutively recruited as cases. For each identified case, two eligible live‐birth deliveries were selected as controls from subsequent deliveries using systematic consecutive sampling until the required 2:1 control‐to‐case ratio was achieved.

Eligibility was verified using delivery registers, clinical records, and laboratory documentation. Written informed consent was obtained in a private setting by trained research staff prior to participation. Participants were assigned unique study identification numbers to ensure confidentiality.

#### 2.2.2. Variables and Measurements

##### 2.2.2.1. Exposure Variables

The primary exposure variable was maternal syphilis infection status at delivery. Syphilis infection was defined as a reactive RPR test confirmed by a reactive treponemal‐specific assay (Treponema pallidum haemagglutination assay, TPHA). Participants with nonreactive RPR results were classified as uninfected. Maternal syphilis infection status at delivery was used as a proxy indicator of antenatal exposure because untreated or inadequately treated infection typically persists throughout pregnancy and remains detectable at the time of delivery. RPR testing was performed by the hospital laboratories at Jinja and Hoima Regional Referral Hospitals as part of routine clinical care, and results were abstracted from laboratory documentation. Testing was therefore site‐based rather than centralized in a single reference laboratory.

Clinical staging of syphilis at the time of testing was not consistently documented and was therefore not included in the primary analysis. Participants were classified according to serologic evidence of infection at delivery.

Maternal RPR titers were categorized into ordinal exposure groups to reflect infection severity and biological gradient:

• low (< 1:4)
• moderate (1:4–1:8)
• high (> 1:8).

These categories were prespecified based on epidemiological evidence demonstrating increasing fetal risk with higher nontreponemal antibody titers.

##### 2.2.2.2. Outcome Variable

The primary outcome variable was stillbirth, defined as fetal death occurring at ≥ 26 weeks of gestation or with a birth weight ≥ 800 g before or during delivery, in accordance with Uganda National Maternal and Newborn Care Guidelines.

##### 2.2.2.3. Covariates and Confounder Selection

Potential confounders included the following:

• maternal age;
• HIV infection status;
• malaria during pregnancy;
• hypertensive disorders of pregnancy;
• antenatal care attendance;
• previous history of stillbirth; and hospital site.

Confounders were selected a priori based on biological plausibility, published epidemiological evidence, and their hypothesized position within the causal pathway linking maternal infection to placental dysfunction and fetal death.

##### 2.2.2.4. Data Collection Procedures

Data were collected between January and March 2026 using standardized data abstraction tools applied to maternity registers, antenatal records, laboratory reports, and delivery records. Maternal syphilis infection status and RPR titers were obtained from hospital laboratory documentation. Information on maternal demographic characteristics and clinical variables was extracted from antenatal and intrapartum records.

Daily supervision of recruitment and data abstraction procedures was conducted by site supervisors to ensure adherence to study protocols and completeness of data collection.

#### 2.2.3. Missing Data Handling

Participants with incomplete laboratory confirmation of syphilis infection status, missing RPR titer measurements, or incomplete covariate information required for multivariable modeling were excluded during eligibility screening. No variables included in the final regression models had missing data exceeding minimal analytical thresholds; therefore, complete‐case analysis was performed.

### 2.3. Sample Size Calculation

Sample size was calculated using the Kelsey method for unmatched case–control studies implemented in Open Epi Version 3. The calculation assumed a 95% confidence level (Zα/2 = 1.96), 80% statistical power (Zβ = 0.84), a control‐to‐case ratio (*r*) of 2:1, an expected proportion of exposure among controls (P2) of 1% (0.01), and an expected proportion of exposure among cases (P1) of 6.3% (0.063), based on prior epidemiological evidence.

The Kelsey formula for unmatched case–control studies is

(1) n1=Zα/2+Zβ2×P1211−P+P12−P/rP12−P2,

where

• *n*_1_ = required number of cases
• *P*1 = proportion exposed among cases
• *P*2 = proportion exposed among controls
• *r* = ratio of controls to cases

Substituting study assumptions:

(2) n1=1.960.84+2×0.0630.937×+0.010.99×/20.0630.01−2,

(3) n1=7.84×0.05900.00495+0.0532,

(4) n1=7.840.06395×0.002809,

(5) n1≈123 cases.

With a 2:1 control‐to‐case ratio:

(6) n2=2123246×= controls.

Thus, the minimum required sample size was 369 participants (123 cases and 246 controls). After accounting for a 10% nonresponse rate and additional requirements for dose–response analysis across maternal RPR titer categories, the final sample size was increased to 406 participants (135 cases and 271 controls).

### 2.4. Data Analysis

Data were entered into secure password‐protected databases and analyzed using statistical software. Descriptive statistics were used to summarize participant characteristics by outcome status.

Differences in the proportion of maternal syphilis infection between stillbirth and live‐birth deliveries were evaluated using the chi‐square test.

Multivariable logistic regression analysis was performed to estimate adjusted odds ratios (AORs) and 95% confidence intervals (CIs) for the association between maternal syphilis infection and stillbirth while controlling for maternal age, HIV status, malaria during pregnancy, hypertensive disorders of pregnancy, antenatal care attendance, previous stillbirth, and hospital site.

Because participants were nested within two referral hospitals, regression models incorporated adjustment for clustering at the hospital level using robust variance estimation to improve the validity of statistical inference.

Maternal RPR titers were analyzed as ordered exposure categories (negative, low, moderate, and high) to evaluate biological gradient effects consistent with Bradford–Hill criteria for causal inference. Trend analysis across ordinal exposure categories was conducted to assess dose–response relationships between increasing antibody titers and stillbirth risk.

Sensitivity analyses were prespecified to assess the robustness of findings, including ordinal trend modeling of RPR titers and evaluation of adjusted associations across exposure strata. Statistical significance was defined using two‐sided tests with a threshold of *p* < 0.05.

All analyses were prespecified except model diagnostics and sensitivity checks, which were conducted to assess robustness of the findings. Categorical variables were summarized as frequencies and percentages, and continuous variables were summarized using means with standard deviations or medians with interquartile ranges, depending on distribution. Chi‐square tests were used to compare categorical variables between stillbirth and live‐birth groups. Multivariable logistic regression was used to estimate crude odds ratios (cORs), AORs, 95% CIs, and *p* values. Robust variance estimation was used to account for clustering by hospital site. Ordinal trend analysis was used to assess dose–response across RPR titer categories. Model fit was assessed using the Hosmer–Lemeshow goodness‐of‐fit test, and multicollinearity was assessed using variance inflation factors. All statistical tests were two‐sided, and statistical significance was set at *p* < 0.05. Analyses were conducted using [STATA version 17].

#### 2.4.1. Bias Control

Selection bias was minimized through consecutive recruitment of all eligible stillbirth cases and systematic selection of controls from subsequent eligible live‐birth deliveries within the same study period. Information bias was reduced through the use of standardized abstraction tools and laboratory‐confirmed exposure classification. Confounding was addressed using multivariable regression adjustment guided by an a priori causal framework.

Reporting of this observational case–control study was guided by applicable items from the STROBE statement for case–control studies.

#### 2.4.2. Ethical Approval and Consent

Ethical approval was obtained from the Kampala International University Research Ethics Committee (KIU‐2025‐1820). The study was registered with the Uganda National Council for Science and Technology, and administrative permissions were obtained from Jinja and Hoima Regional Referral Hospitals. Written informed consent was obtained from all participants before enrolment. For participants below the legal age of independent consent, assent and guardian permission were obtained as applicable.

## 3. Results

### 3.1. Characteristics of the Study Participants

A total of 406 women were included in the analysis, comprising 271 women with live‐birth deliveries and 135 women with stillbirth deliveries. Maternal age and number of antenatal care visits were approximately normally distributed, with a mean age of 28.1 ± 6.0 years and mean antenatal visits of 3.9 ± 1.8. Gravidity, parity, gestational age at delivery, and birth weight were not normally distributed and were summarized using medians and interquartile ranges.

Compared with women with live‐birth deliveries, women with stillbirth deliveries had a higher proportion aged 40 years or older (6.7% versus 0.7%), lower attendance of four or more antenatal care visits (48.1% versus 66.4%), more frequent third‐trimester antenatal care initiation (51.1% versus 30.3%), and a higher prevalence of maternal syphilis infection (11.9% versus 3.7%). Education level and gravidity also differed significantly between the two outcome groups. Residence, parity, previous stillbirth, history of genital ulcers, HIV status, malaria in pregnancy, hypertensive disorder of pregnancy, and hospital site did not differ significantly between live‐birth and stillbirth groups (Table 1).

**TABLE 1:** Descriptive characteristics of participants by birth outcome (*N* = 406).

Characteristic	Category	Live birth *n* = 271 *n* (%)	Stillbirth *n* = 135 *n* (%)	Overall *N* = 406 *n* (%)	*χ* ^2^(df), *p* value
Maternal age	< 20	26 (9.6)	7 (5.2)	33 (8.1)	*χ* ^2^(3) = 16.169, *p* = 0.001
	20–29	151 (55.7)	64 (47.4)	215 (53.0)	
	30–39	92 (33.9)	55 (40.7)	147 (36.2)	
	40+	2 (0.7)	9 (6.7)	11 (2.7)	

Residence	Urban	117 (43.2)	48 (35.6)	165 (40.6)	*χ* ^2^(1) = 1.863, *p* = 0.172
	Rural	154 (56.8)	87 (64.4)	241 (59.4)	

Education level	No formal	9 (3.3)	9 (6.7)	18 (4.4)	*χ* ^2^(3) = 10.264, *p* = 0.016
	Primary	86 (31.7)	52 (38.5)	138 (34.0)	
	Secondary	122 (45.0)	62 (45.9)	184 (45.3)	
	Tertiary	54 (19.9)	12 (8.9)	66 (16.3)	

Gravidity group	Primigravida	74 (27.3)	21 (15.6)	95 (23.4)	*χ* ^2^(1) = 6.302, *p* = 0.012
	Multigravida	197 (72.7)	114 (84.4)	311 (76.6)	

Parity group	P1 or less	237 (87.5)	114 (84.4)	351 (86.5)	*χ* ^2^(1) = 0.464, *p* = 0.496
	Multipara	34 (12.5)	21 (15.6)	55 (13.5)	

Previous stillbirth	No	246 (90.8)	117 (86.7)	363 (89.4)	*χ* ^2^(1) = 1.202, *p* = 0.273
	Yes	25 (9.2)	18 (13.3)	43 (10.6)	

ANC attendance	< 4	91 (33.6)	70 (51.9)	161 (39.7)	*χ* ^2^(1) = 11.821, *p* ≤ 0.001
	4+	180 (66.4)	65 (48.1)	245 (60.3)	

Timing of first ANC visit	No ANC	8 (3.0)	8 (5.9)	16 (3.9)	*χ* ^2^(3) = 27.920, *p* ≤ 0.001
	First trimester	56 (20.7)	7 (5.2)	63 (15.5)	
	Second trimester	125 (46.1)	51 (37.8)	176 (43.3)	
	Third trimester	82 (30.3)	69 (51.1)	151 (37.2)	

History of genital ulcers	No	251 (92.6)	120 (88.9)	371 (91.4)	*χ* ^2^(1) = 1.154, *p* = 0.283
	Yes	20 (7.4)	15 (11.1)	35 (8.6)	
HIV status	Negative	244 (90.0)	117 (86.7)	361 (88.9)	*χ* ^2^(1) = 0.725, *p* = 0.395
	Positive	27 (10.0)	18 (13.3)	45 (11.1)	

Malaria in pregnancy	No	234 (86.3)	110 (81.5)	344 (84.7)	*χ* ^2^(1) = 1.294, *p* = 0.255
	Yes	37 (13.7)	25 (18.5)	62 (15.3)	

Hypertensive disorder of pregnancy	No	241 (88.9)	119 (88.1)	360 (88.7)	*χ* ^2^(1) = 0.005, *p* = 0.946
	Yes	30 (11.1)	16 (11.9)	46 (11.3)	

Maternal syphilis status	Negative	261 (96.3)	119 (88.1)	380 (93.6)	*χ* ^2^(1) = 8.699, *p* = 0.003
	Positive	10 (3.7)	16 (11.9)	26 (6.4)	

Hospital site	Jinja RRH	145 (53.5)	77 (57.0)	222 (54.7)	*χ* ^2^(1) = 0.322, *p* = 0.570
	Hoima RRH	126 (46.5)	58 (43.0)	184 (45.3)	

*Note:* Percentages are column percentages. *p* values were derived from chi‐square tests comparing live‐birth and stillbirth groups.

Abbreviation: ANC, antenatal care.

#### 3.1.1. Proportion of Maternal Syphilis Infection by Birth Outcome

**FIGURE 1:**
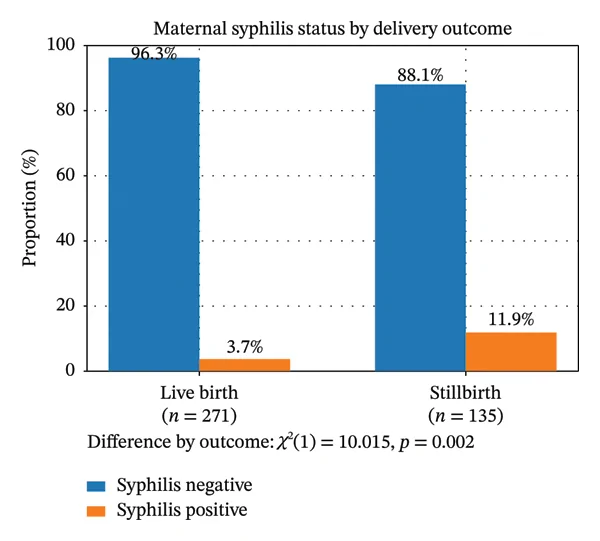
Proportion of maternal syphilis infection among stillbirth versus live‐birth deliveries.

As shown in Figure 1, maternal syphilis infection was more common among stillbirth deliveries than among live‐birth deliveries at Jinja and Hoima Regional Referral Hospitals. After adjustment for important maternal and obstetric confounders, maternal syphilis infection was independently associated with higher odds of stillbirth.

#### 3.1.2. Association Between Maternal Syphilis Infection and Stillbirth

Maternal syphilis infection was independently associated with stillbirth. In crude analysis, women with maternal syphilis infection had higher odds of stillbirth than women without syphilis infection (cOR 3.51, 95% CI: 1.55–7.96; *p* = 0.003). After adjustment for maternal age group, HIV status, malaria in pregnancy, hypertensive disorder of pregnancy, antenatal care attendance, previous stillbirth, and hospital site, maternal syphilis infection remained independently associated with stillbirth (AOR 3.91, 95% CI: 1.65–9.31; *p* = 0.002) (Table 2).

**TABLE 2:** Association between maternal syphilis infection and stillbirth, *N* = 406.

Maternal syphilis infection	Live birth *N* = 271 *n* (%)	Stillbirth *N* = 135 *n* (%)	cOR	95% CI	*p* value	aOR	95% CI	*p* value
Negative	261 (96.3)	119 (88.1)	Ref			Ref		
Positive	10 (3.7)	16 (11.9)	3.51	1.55–7.96	0.003	3.91	1.65–9.31	**0.002**

*Note:* Percentages are column percentages. Ref: reference category. Bold value differentiates significant values from those not significant.

Abbreviations: ANC, antenatal care; aOR, adjusted odds ratio; CI, confidence interval; cOR, crude odds ratio; HIV, human immunodeficiency virus.

The multivariable model was statistically significant overall (LR *χ* ^2^ (10) = 44.07, *p* < 0.001), with a pseudo‐R^2^ of 0.0854. The Hosmer–Lemeshow goodness‐of‐fit test showed no evidence of poor fit (*χ* ^2^ (8) = 4.36, *p* = 0.823). Using a probability cut‐off of 0.5, the model correctly classified 70.9% of observations. Multicollinearity assessment showed no evidence of problematic collinearity among covariates, with a mean variance inflation factor of 1.56.

#### 3.1.3. Dose–Response Relationship Between RPR Titer and Stillbirth

**FIGURE 2:**
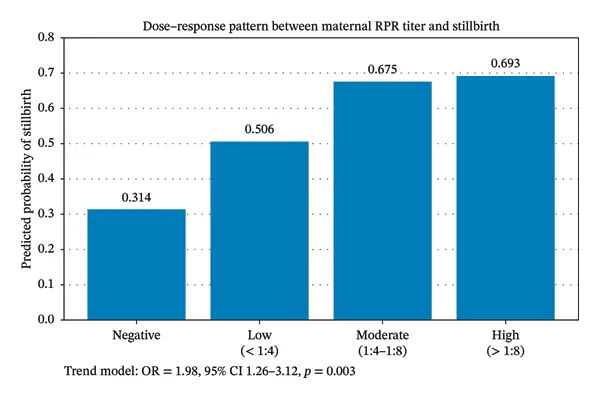
Dose–response bar chart showing predicted probability of stillbirth across maternal RPR titer categories.

A dose–response pattern was observed between increasing RPR titer category and stillbirth (Table 3 and Figure 2). The predicted probability of stillbirth increased from 0.314 among women without maternal syphilis to 0.506 among women with low titers, 0.675 among women with moderate titers, and 0.693 among women with high titers.

**TABLE 3:** Dose–response relationship between maternal RPR titer category and the odds of stillbirth.

RPR titer category	Live birth *n* (%)	Stillbirth *n* (%)	cOR	95% CI	*p* value	aOR	95% CI	*p* value
Negative	261 (96.3)	119 (88.1)	Ref			Ref		
Low (< 1:4)	5 (1.9)	6 (4.4)	2.63	0.80–8.79	0.116	2.38	0.66–8.63	0.186
Moderate (1:4–1:8)	3 (1.1)	5 (3.7)	3.66	0.86–15.55	0.079	5.05	1.13–22.68	0.035
High (> 1:8)	2 (0.7)	5 (3.7)	5.48	1.05–28.67	0.044	5.48	0.97–30.79	0.054
p‐trend						1.98	1.26–3.12	0.003

*Note:* Percentages are column percentages. Ref: reference category.

Abbreviations: ANC, antenatal care; CI, confidence interval; HIV, human immunodeficiency virus; OR, odds ratio.

Compared with women without maternal syphilis, women with moderate RPR titers had about five times higher adjusted odds of stillbirth (AOR 5.05, 95% CI: 1.13–22.68; *p* = 0.035). Women with high RPR titers had similarly elevated odds of stillbirth, although the CI was wide and the association was borderline statistically significant (AOR 5.48, 95% CI: 0.97–30.79; *p* = 0.054). Low titers were not significantly associated with stillbirth (AOR 2.38, 95% CI: 0.66–8.63; *p* = 0.186).

The ordinal trend model confirmed a statistically significant dose–response relationship: each stepwise increase in RPR titer category nearly doubled the odds of stillbirth (OR 1.98, 95% CI: 1.26–3.12; *p* = 0.003). The RPR titer model was statistically significant overall (LR *χ* ^2^ (11) = 42.79, *p* < 0.001), with no evidence of poor model fit on Hosmer–Lemeshow testing (*χ* ^2^ (8) = 7.13, *p* = 0.523).

## 4. Discussion

### 4.1. Proportion of Maternal Syphilis Infection Among Stillbirth Deliveries

Maternal syphilis infection was proportionally more frequent among women delivering stillborn infants than among those delivering live‐born infants in this study. This disproportionate distribution indicates that maternal syphilis infection remains an important contributor to fetal mortality in referral‐hospital settings in Uganda. Similar patterns have been reported across multiple sub‐Saharan African referral settings. For example, a prospective case–control study in Malawi reported that untreated early‐stage maternal syphilis was more prevalent among pregnancies complicated by adverse birth outcomes, including stillbirth, compared with healthy term deliveries, with an odds ratio of 7.13 for adverse outcomes among untreated infections [7]. In Tanzania, cross‐sectional hospital‐based evidence further demonstrated a measurable burden of maternal syphilis among women presenting with macerated stillbirth, with seropositivity significantly associated with HIV co‐infection and rural residence—both markers of delayed access to antenatal screening services [6]. Regional systematic reviews synthesizing African observational studies similarly report consistently elevated odds ratios ranging from approximately 2.07 to 16.83 for stillbirth among women with untreated maternal syphilis compared with seronegative women [7].

The consistency of these findings across multiple African referral and district hospital populations supports the interpretation that maternal syphilis infection remains an important contributor to stillbirth burden in high‐transmission settings where antenatal screening coverage remains incomplete or delayed. These regional comparisons strengthen the external validity of the present findings within East African referral‐hospital populations.

### 4.2. Association Between Maternal Syphilis Infection and Stillbirth

After adjustment for maternal age, HIV infection, malaria in pregnancy, hypertensive disorders of pregnancy, antenatal care attendance, previous stillbirth, and hospital site, maternal syphilis infection remained independently associated with nearly fourfold higher odds of stillbirth. This magnitude of association is consistent with findings from regional observational studies demonstrating markedly elevated stillbirth risk among women with untreated or active maternal syphilis infection. For example, a retrospective cohort study in Tanzania reported a stillbirth rate of 25% among women with untreated high‐titer syphilis compared with 1% among seronegative women, corresponding to an adjusted risk ratio of 18.1 after controlling for maternal age, gravidity, HIV infection, malaria, and anemia [14]. Similarly, a prospective case–control study in Malawi reported elevated odds of adverse birth outcomes—including stillbirth—among untreated maternal infections even after adjustment for major maternal demographic and clinical factors [5].

Evidence from systematic reviews further demonstrates that untreated maternal syphilis substantially increases the probability of fetal loss, with pooled estimates indicating stillbirth or early fetal death occurring in approximately one‐quarter of untreated infected pregnancies compared with fewer than 5% among uninfected women [3]. These findings reinforce the interpretation that maternal syphilis infection represents a strong and independent determinant of stillbirth risk in referral‐hospital populations in sub‐Saharan Africa.

The persistence of the association after adjustment for HIV infection, malaria in pregnancy, hypertensive disorders, and antenatal care attendance strengthens confidence that the observed relationship is unlikely to be explained solely by confounding from other infectious or obstetric risk factors common in this setting.

### 4.3. Dose–Response Relationship Between Maternal RPR Titers and Stillbirth

This study demonstrated a graded increase in stillbirth risk across increasing maternal RPR titer categories, with moderate and high titers associated with substantially higher adjusted odds of stillbirth compared with women without syphilis infection. The ordinal trend analysis further showed that each stepwise increase in RPR titer category was associated with nearly doubled odds of stillbirth, providing strong evidence of a biological gradient.

The presence of a dose–response relationship between increasing antibody titers and stillbirth risk is consistent with findings from large PMTCT program datasets and multicenter observational studies demonstrating that maternal titers greater than 1:4 significantly increase the probability of stillbirth and other adverse pregnancy outcomes even after multivariable adjustment for maternal demographic and clinical characteristics [8]. Similarly, cohort analyses have demonstrated that each twofold increase in maternal non‐treponemal antibody titers is associated with progressively increasing odds of adverse pregnancy outcomes, reflecting increasing maternal spirochetemia and placental infection risk [9, 15].

Population‐based registry analyses from Brazil further demonstrate progressive increases in adverse pregnancy outcomes—including preterm birth and small‐for‐gestational‐age infants—across increasing maternal VDRL titer categories, supporting the biological plausibility of a graded relationship between infection severity and fetal compromise [16].

From a causal inference perspective, the demonstration of a biological gradient in the present study satisfies one of the Bradford–Hill criteria supporting causality and suggests that maternal RPR titers may provide clinically useful information for risk stratification in antenatal care settings in high‐burden referral‐hospital populations.

#### 4.3.1. Interpretation of Findings in the Context of Referral‐Hospital Settings

Referral hospitals in Uganda serve large catchment populations that often include women with delayed antenatal care initiation, untreated infections, and multiple obstetric risk factors. The independent association between maternal syphilis infection and stillbirth observed in this study therefore has important implications for strengthening screening and treatment strategies in high‐volume tertiary‐care settings. The identification of increasing stillbirth risk across RPR titer categories further suggests that infection severity may help guide prioritization of clinical management and surveillance during pregnancy.

### 4.4. Study Strengths and Limitations

This study has several strengths. It used laboratory‐based syphilis ascertainment, included both stillbirth cases and live‐birth controls from two referral hospitals, and adjusted for clinically important confounders including HIV, malaria, hypertensive disorders, antenatal care attendance, previous stillbirth, and hospital site. It also evaluated RPR titer categories, allowing assessment of a dose–response relationship rather than relying only on binary infection status.

The study also has limitations. The hospital‐based case–control design may limit generalizability to women delivering outside referral hospitals. Residual confounding from unmeasured factors, including other infections, congenital anomalies, socioeconomic status, and nutritional factors, cannot be excluded. Information on timing of maternal infection, clinical stage, timing and completeness of treatment, partner treatment, serofast status, and repeat testing during pregnancy was incomplete. Consequently, treated or partially treated infections could not be reliably distinguished from untreated active infection, which may have influenced the observed associations. Because RPR testing was performed in routine site laboratories rather than a single central laboratory, interlaboratory or observer variability in titer interpretation cannot be fully excluded. In addition, other infectious causes of fetal compromise, including cytomegalovirus, parvovirus B19, hepatitis E, and rubella, were not routinely tested and may have contributed to some stillbirths.

### 4.5. Conclusion

Maternal syphilis infection was more common among stillbirth deliveries than among live‐birth deliveries at Jinja and Hoima Regional Referral Hospitals. After adjustment for important maternal and obstetric confounders, maternal syphilis infection was independently associated with substantially higher odds of stillbirth.

Increasing RPR titer categories showed a dose–response relationship with stillbirth. These findings support strengthened antenatal syphilis screening, timely treatment, partner management, and risk‐based follow‐up, while recognizing that the hospital‐based design and incomplete treatment‐history data limit causal interpretation.

### 4.6. Recommendations

We recommend that the Ministry of Health and district health teams strengthen universal antenatal syphilis screening, ensure same‐visit treatment of all infected pregnant women, and maintain reliable supply chains for rapid diagnostic tests and benzathine penicillin. Health workers should document RPR titers, repeat testing where clinically indicated, treatment timing, and partner notification or treatment. Moderate or high titers should prompt urgent treatment, closer fetal surveillance, and careful follow‐up. Hospital stillbirth review teams should include antenatal syphilis testing, treatment timing, RPR titer documentation, partner management, and post‐stillbirth family counselling in routine perinatal death review.

**Nomenclature**
ANC: antenatal care
HIV: Human immunodeficiency virus
KIU‐REC: Kampala International University Research Ethics Committee
RPR: Rapid plasma reagin
STI: Sexually transmitted infection
TPHA: Treponema pallidum haemagglutination assay
TPPA: Treponema pallidum particle agglutination assay
UNCST: Uganda National Council for Science and Technology

## Author Contributions

Daniel Mugabi developed the proposal and participated in data collection and analysis. Theoneste Hakizimana, Musa Kasujja, and Ronard Musinguzi had significant contribution in data collection and analysis and drafting of the manuscript. Adam Eibad, Geoffrey Okot, John Yaweh, Sande George Bina, Neel Rajendra, Emmanuel Okurut, and Dominic Byarugaba participated in making corrections of the proposal and analysis process.

## Funding

This research received no specific grant from any funding agency in the public, commercial, or not‐for‐profit sectors.

## Disclosure

Ronard Musinguzi affirms that this manuscript is an honest, accurate, and transparent account of the study being reported; that no important aspects of the study have been omitted; and that any discrepancies from the study as planned, and where relevant registered, have been explained. All authors have read and approved the final version of the manuscript. Ronard Musinguzi had full access to all of the data in this study and takes complete responsibility for the integrity of the data and the accuracy of the data analysis. No funding source had any role in the study design; collection, analysis, or interpretation of data; writing of the manuscript; or the decision to submit the manuscript for publication.

## Ethics Statement

Ethical approval was obtained from the Kampala International University Research Ethics Committee (KIU‐2025‐1820). The study was registered with the Uganda National Council for Science and Technology, and administrative permissions were obtained from Jinja and Hoima Regional Referral Hospitals. Written informed consent was obtained from all participants, with assent and guardian permission where applicable.

## Conflicts of Interest

The authors declare no conflicts of interest.

## Data Availability

The data supporting the findings of this study are available from Kampala International University Western Campus library and from the corresponding author upon reasonable request, subject to applicable institutional and ethical requirements.
